# A Nutrition Education Intervention to Combat Undernutrition: Experience from a Developing Country

**DOI:** 10.5402/2013/210287

**Published:** 2013-02-05

**Authors:** Ayesha Zahid Khan, Ghazala Rafique, Haneen Qureshi, Salma Halai Badruddin

**Affiliations:** ^1^Human Development Programme, Aga Khan University, METRO Cash & Carry Pakistan (Pvt) Ltd., Mezzanine Floor, Main University Road, Karachi 75300, Pakistan; ^2^Mahar Medical Center, F-50 A, Off 26th Street, Block 4, Clifton, Karachi 75500, Pakistan

## Abstract

*Introduction.* Undernutrition in children is a major public health concern in Pakistan. A number of interventions which focused only on providing nutrient supplementation have failed to change child undernutrition status during the last 2 decades. The present study aimed to assess the impact of nutrition education on the nutritional status of children living in resource-limited environments. *Methods.* Subjects were 586 children from Tando Jam and Quetta, Pakistan, aged from 6 months to 8 years. Children were characterized as mild, moderate, or severely wasted on Z-scores. Anthropometry and 24-hour dietary recall were used for nutritional assessment. Intervention strategy was nutrition counselling targeting mothers. Primary outcome was decrease in the severity of wasting and changes in the feeding practices. *Results*. Nearly 36% children in Tando Jam and 32% children in Quetta progressed to a normal nutritional status. There was a significant increase in the number of meals taken per day (Tando Jam—*P* ≤ 0.000/Quetta—*P* ≤ 0.025). In Tando Jam, significant increase was reported in the intake of high starch food items, vegetables, and fruits (*P* ≤ 0.000). In Quetta, significant increase was noted in the intake of plant protein (*P* ≤ 0.005), dairy foods (*P* ≤ 0.041), and vegetables (*P* ≤ 0.026). *Conclusion*. Nutrition education was successful in reducing undernutrition in food insecure households.

## 1. Introduction

In Pakistan, undernutrition is a major public health concern and an important underlying factor for the high infant and under 5 mortality rate. Undernutrition is common among all sections of the Pakistani population, but is of greatest consequence in young children. The country has one of the highest rates of infant mortality in South Asia (61.27 deaths/1,000 live births) [1]. The findings of Pakistan demographic and health survey 2006-2007 highlight the fact that the country's rate of infant and child mortality has hardly changed in over a decade [2] and the undernutrition status has remained unchanged for almost two decades [3]. The National Nutrition Survey (NNS) of Pakistan (2011) reports malnutrition estimates of less than −2 Z-scores in children under five years of age as underweight 31.5%, wasting 15%, and stunting 43.7% [3]. The NNS-2011 also reported childhood undernutrition levels to be higher in rural areas with the risk rising significantly for children between 12 and 35 months of age and for the children of older mothers, whereas the risk appears lower for children given food earlier than one year. 

There is no doubt that poverty is an important risk factor for undernutrition in Pakistan, but lack of economic resources is not the only constraint. Other major contributing factors are lack of awareness regarding the importance of breast feeding [4], improper complimentary feeding practices [5], and early or delayed introduction of complementary foods [6]. A cross-sectional study undertaken in Peshawar concludes that the majority of women do not give colostrum to their babies, more than 50% of mothers do not start weaning at the recommended age of six months and the majority of the children were fed commercial weaning formulas [7]. Delayed introduction and inadequate quantity of complimentary food have also been reported as contributing factors for undernutrition [6]. Diarrhea appears to be a significant contributor indicating sanitation to be an important factor for reducing the risk of undernutrition [8].

The Human Development Programme (HDP) of Aga Khan University (AKU) implemented a community-based Early Child Development Parenting Programme in Tando Jam, Sindh, and Quetta, Balochistan from 2005 to 2011. The parenting programme was primarily geared towards improvement of child nurture by provision of appropriate information and development of skills of mothers/caregivers and their families to improve child wellbeing in the early years (0–8 years). The key service delivery of this programme was Early Child Development (ECD) and parenting education that was done through regular home visits by ECD Workers (ECDWs) who were young local women trained in early childhood development assessment, child care, and nurture. Children aged 0–12 months were visited every month and children aged 1–8 years were visited every three months. During each visit the weight and length/height of the children were taken. Also, there were an assessment of psychomotor development of children aged 0–3 years and a consult with mothers regarding nutrition, health and hygiene, child rearing practices, and provision of age-appropriate stimulation and learning opportunities to enhance child development.

The field anthropometry data analysed revealed an overall wasting rate of 26.7% in the children on the two field sites (Tando Jam 29.4%; Quetta 22.5%). Based on the field data, a careful pilot nutrition intervention program which included 40 children was designed and implemented. During this phase, the ECDWs were extensively trained in assessing dietary patterns through 24-hour dietary recall (24 HDR) and to provide counselling based on the findings. The pilot provided guidance in forming a major intervention strategy to combat under nutrition at both field sites.

The present study aimed to improve the nutritional status of children living in resource-limited environment through nutrition education. The hypothesis was that nutrition education can improve nutritional status of children. This study reports the results of a three-month intervention (October–December, 2010) on the change in nutritional status of undernourished children living in two areas of Pakistan.

## 2. Methods

### 2.1. Study Participants

Study participants were from HDP field sites of Tando Jam, Sindh and Quetta, Balochistan, in Pakistan. The 586 children (Tando Jam: 323; Quetta: 263) targeted for this intervention were between 6 months and 8 years of age. Children were characterized as mild, moderate or severely wasted on Z-scores. The intervention strategy focused on nutrition education based on individual counselling sessions targeting the mothers. The majority of these women could not read and write and so the counseling sessions were comprised of verbal discussions. Direct counselling sessions were also conducted with undernourished children of the age 5 years and older. 

### 2.2. Primary Outcome

The primary outcome was decrease in the severity of wasting (−2 Z-score weight for length) as well as changes in feeding practices of the care givers. 

### 2.3. The Nutrition Intervention

The ECDWs received structured training to assess dietary patterns through 24 HDR and to provide nutrition counselling based on the dietary assessment. Where needed, individual cases were referred to the program nutritionist and dietary advice was further modified according to individual family circumstances. Two nutrition education booklets in Urdu language were prepared to reenforce training [10]. The focus of nutrition messages and advice was as follows.

Children aging from 6 months to 1 year.

- Start complimentary foods at six months of age.
- Continue breastfeeding till two and a half years of age.
- Use home-cooked local, simple foods like *khitchri* (a mixture of cooked rice and lentils) and milk desserts like *kheer *and *firni* (sweet dish of milk and rice).
- Include soft fruits and cooked vegetables in diet.
- The Child should be offered food/breast milk at least 5-6 times in a day.

Children aging from 1 year to 8 years.

- Continue giving the child 5-6 meals in a day.
- Advice mothers to give more importance to the child during meal times.
- Add protein food to meals (use plant as well as animal protein sources).
- Encourage milk/yogurt intake.
- Discourage tea intake in children.
- Discourage use of food items with low nutrient density such as chips, sweets and beetle nut.
- Encourage children to use snack money to buy locally available fruits, vegetables, and nuts rather than chips, sweets, and beetle nut.
- After five years of age, counsel the child directly along with the mother.

### 2.4. Visit Protocol

The first month visiting protocol was defined on the basis of severity of wasting on Z-scores. The Z-scores lines on a growth chart indicate distance from the median. The ECDW visiting protocol was as the folowing:

- severely wasted (<−3 Z-score): weekly,
- moderately wasted (−2 to ≥−3 Z-score): fortnightly,
- mildly wasted (−1 to ≥−2 Z-score): monthly.

After the first month all children were visited once per month only. 

### 2.5. Data Collection

The anthropometry data was collected for every enrolled child at every visit by the ECDWs who were trained to take accurate measurements. Length of children aged less than 2 years was measured by infant length scale. Height was taken by leister scale (SECA 213, Portable Height Measure). Weight for younger children was taken using the digital Baby Weighing Scale (Laica, Italy, BF-2051), while for children weighing above 20 kg the Digital Bathroom Scale (Beurer-PS07-Max 150 kg) was used. An instruction manual had been specially developed in Urdu for taking these measurements [11]. The World Health Organization (WHO) growth reference charts were used to plot growth progress of each child [12]. The anthropometry assessment helped to discuss the child's progress with the mothers.

The 24 HDR data was collected on forms that had been previously piloted in the community. These forms were checked by field supervisors for completion and later edited and entered at the head office.

## 3. Statistical Analysis

The final analysis was run on 586 (Tando Jam *n* = 323, Quetta *n* = 263). The data of 10 children was removed from analysis because their anthropometry or 24 HDR was missing. The reason for missing data was either the families had migrated out of the area or the family had refused to have the child measured. The anthropometry and 24 HDR data of baseline and end of month 3 were used for analysis. 

Dual entry and cleaning of data were done on Epidata (V 3.1). Height/length and weight were analysed by WHO-Anthro [13] to calculate severity of wasting.For children who were under 5 years of age, WHO software (V2.0.4) was used. For children over 5 years, WHO syntax (2007) file was used. Data was analysed on SPSS version 13. After running, descriptive and cross-tabs significance was tested by Kendall's tau-c. 

The 24 HDR was analysed for the number of servings from each food group. Raw data was converted into categories and categories were entered in MS Excel. Dual check was done in EpiData (V 3.1). Data was analyzed in SPSS (V 13). After running descriptive and cross-tabs *t*-test was run for significance. Data appeared skewed so a nonparametric test, the Wilcoxon, was run for significance. For details of the serving sizes and description of the food items, see Box 1.

## 4. Results

The sociodemographic characteristics of the intervention group are described in Table 1. The 586 children (Tando Jam—323, Quetta—263) targeted for this undernutrition intervention were between 6 months to 8 years of age. The number of boys was Tando Jam: 170 (52.6%) and Quetta: 125 (47.5%). The number of girls was Tando Jam: 153 (47.4%) and Quetta: 138 (52.5%). Most of the mothers were young (31-32 years of age) and were not pursing jobs outside home. At the Tando Jam site 53.4% mothers and at Quetta site only 23.3% mothers had received formal education. Some households had more than one undernourished child. There were no significant differences between the sociodemographic characteristics of the two field sites.

**Figure 1:**
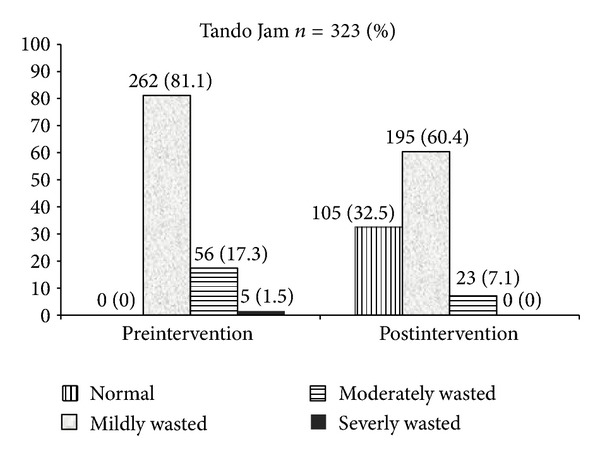
Pre- and postintervention malnutrition status (wasting) of study participants, Tando Jam, *n* = 323 (%).

**Figure 2:**
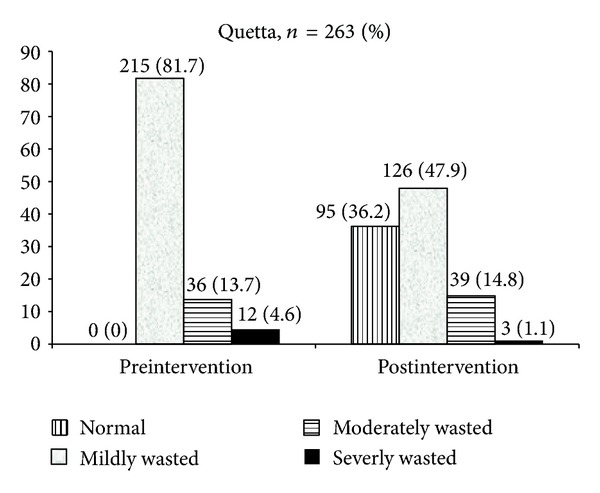
Pre- and postintervention malnutrition status (wasting) of study participants, Quetta, *n* = 263 (%).

Impact of the nutrition education intervention on the nutritional status of the children, with wasting taken as the outcome parameter, is shown in Figures 1 and 2. At baseline of both field sites the majority of the undernourished children were categorized as mildly wasted (−1 to ≥−2 Z-score). The largest impact of the intervention was seen on this group. In Tando Jam the prevalence of mildly wasted dropped from 81% to 60%. In Quetta, the impact was stronger with the number of mildly wasted reducing from 82% to 49%. Although the number of severely wasted children was small at both the site (Tando Jam: 5, Quetta: 12), this number further dropped to 0 in Tando Jam and to 3 in Quetta. There was no difference amongst boys and girls in reduction of wasting patterns.

Tables 2 and 3 denote the changes in dietary practices of the target group in Tando Jam and Quetta reportedly. At both field sites there was a significant increase in the number of meals per day taken by the children (Tando Jam-*P* ≤ 0.000, Quetta-*P* ≤ 0.025). In Tando Jam the most significant increase was in the intake of high starch food items (*P* ≤ 0.000). Significant increase was additionally reported in the intake of vegetables (*P* ≤ 0.000) and fruits (*P* ≤ 0.000). However in Quetta a significant increase was noted in the intake of plant protein (*P* ≤ 0.005), dairy foods (*P* ≤ 0.041), and vegetables (*P* ≤ 0.026). 

## 5. Discussion

Our intervention was successful in reducing undernutrition at both ECD field sites. Nearly 36% children in Tando Jam and 32% children at Quetta progressed to a normal nutritional status. Results suggest that poor households within their given resources without food provision can improve growth if specific nutrition education based on personal dietary assessment is provided to them. These findings support evidence from experiences of the region that there is scope for improving feeding behaviour and growth through counselling, education, and behaviour change [14]. Other low income communities have shown that nutrition education delivered through health services can decrease the prevalence of malnutrition and stunting in childhood [15].

Our community-based intervention made the biggest impact on the mildly wasted group. The number of the severely wasted was reduced to nil in the Tando Jam group. The statistics of severely wasted in Quetta show a reduction but in reality all except one of those who were severely wasted at baseline progressed to the moderate or mildly wasted status. Of the 3 severely undernourished children with postintervention in Quetta, we were not able to make a change in the nutritional status of 1 child. Also, 1 child from mild and another from the moderately wasted statuses declined to severe wasting. Previous experience in Pakistan has shown that community-based education interventions, conducted by workers of limited education, can be effective. The Hala perinatal trial was successful in significantly reducing still births and neonatal mortality in a rural area of Pakistan [16]. The trial trained public sector community health workers in rural Pakistan to deliver a package of preventive and promotive health care messages to community members. The focus of community education and advocacy was for facility births and prevention of perinatal and all cause neonatal mortality. Although the overall coverage achieved by the intervention was low, the effect on crucial household behaviors and care seeking patterns was promising. The findings evidence effectiveness of community-based approaches to address newborn mortality in difficult-to-reach areas and support the use of strategies involving outreach workers in such settings. 

We were able to bring about positive changes in dietary practices of the target group with no change in family financial resources. Behaviour Change Communication (BCC) was used to make counselling more effective. Suggestions were specific to household and their practices. At both field sites, the number of meals taken daily by the children significantly increased. Mothers were made aware of the fact that children have small appetites and intake per meal is limited. In order to increase calorie intake, children should be given two healthy snacks besides the three regular meals of the day. In Tando Jam the most significant increase was in the intake of high starch food items. In addition to this, there was significant increase in vegetables and fruits. In Quetta there was a significant increase in the intake of plant protein, dairy products and vegetables. A review of community-based intervention trials in developing countries, undertaken by Bhutta et al., concludes that it is possible to improve child feeding practices through culturally appropriate behaviour change communication techniques [17]. Another review indicates that there is sufficient evidence for implementing promotion of feeding through individual or group counselling and behaviour change communication for improved complementary feeding in food secure areas. However, there is a recommendation for food supplementation in food insecure areas [18]. In Karachi, a community intervention delivered by lay workers trained to counsel for and influence behaviour change was able to bring about positive change in behaviour by delivering focused nutrition education to each house hold in the community [19]. 

Our intervention supports the concept that though poverty may be one factor of undernutrition, nutrition education can be effective in making families aware of the importance of a healthy diet. Families can improve their nutritional health within the resources available to them. In recent times, one of the most outstanding success stories for reducing child malnutrition is the Thailand experience [20]. The success of this intervention is attributed not only to the rapid economic growth of the country but also to the implementation of nutrition programs by Thai government. During 1980–1986, child malnutrition rate was reduced from 50% to 25%. The World Bank report of 2002 quotes the independent survey data for reductions in PEM showing that Thailand reduced moderate malnutrition from about 25% in the under-five population in 1986 to about 15% in 1995 (NCHS standards, <−2SD from the mean, weight for age), thus almost eliminating PEM as a national public health problem. Thailand's success in reducing PEM is therefore unequivocal [20]. The Thai nutrition programs included nutrition information, education, and communication that emphasized increasing food and nutrition knowledge during pregnancy and lactation, promotion of breast feeding, introduction of proper supplementary foods, increased awareness of the five food groups, food hygiene, and correction of false food beliefs and taboos. Production of nutritious foods in communities was also promoted through such activities as home gardening, growing of fruit trees, cultivation of legumes and sesames, fish ponds, and the prevention of epidemic diseases in chicken.

The limitation of this study is that we did not follow up the group to assess the long-term change in nutritional status or dietary habits of the study population. 

It is recommended that this approach should be applied in a lager representative sample to test if nutrition education can improve nutritional status of children in Pakistan. National level programs like the Lady Health Workers program should be expanded to include trained personnel to provide nutrition education. 

## 6. Conclusion

Our intervention was successful in reducing undernutrition at the ECD field sites in two areas of Pakistan. Nearly 36% and 32% children progressed to a normal nutritional status at the respective field sites Tando Jam and Quetta. Results suggest that poor households within their given resources without any food provision can improve growth if specific nutrition education based on personal dietary assessment is provided to them. These findings support the evidence that there is scope for improving feeding behaviour and growth through counselling, nutrition education, and behaviour change communication.

## Figures and Tables

**Box 1:**
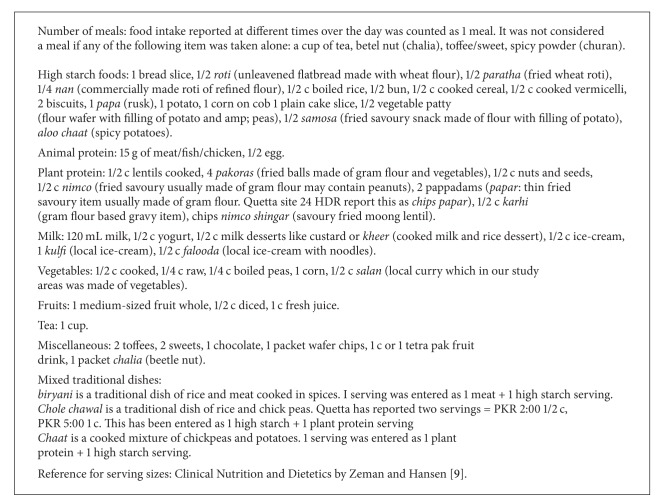
24-hour dietary recall, description of food groups, and serving sizes taken for children.

**Table 1:** Socio-demographic characteristics of the intervention households.

Family information	Tando Jam (*n* 256) Mean (SD)	Quetta (*n* 215) Mean (SD)
Family members	6.2 (2.1)	9.0 (4.5)
Family income (PKR)*	13,970 (9,521)	14,592 (19,972)
Age of mother (years)	32.2 (5.5)	31.2 (5.8)

Mother's school education	Tando Jam *n* 256	Quetta *n* 215
	*n* (%)	*n* (%)

No schooling	117 (45.7)	165 (76.7)
Schooling:		
Primary (1–7)	44 (17.2)	17 (7.9)
Middle (8–10)	53 (20.7)	16 (7.5)
College/University	42 (16.4)	17 (7.9)

*PKR: Pakistani Rupee.

**Table 2:** Change in dietary intake of children aged from 6 months to 8 years, Tando Jam (*n *323).

Number of servings	Preintervention mean (SD)	Postintervention mean (SD)	*P* value
Number of meals	5.42 (1.20)	6.01 (1.19)	0.000
High starch foods	5.87 (1.99)	6.85 (2.38)	0.000
Animal protein	0.92 (1.14)	1.10 (1.47)	0.158
Plant protein	0.77 (1.01)	0.71 (1.03)	0.510
Dairy foods	1.66 (1.93)	1.69 (1.76)	0.417
Vegetables	0.42 (0.70)	0.66 (0.83)	0.000
Fruits	0.51 (0.90)	0.78 (0.94)	0.000
Tea	1.48 (0.64)	1.51 (0.63)	0.433
Miscellaneous	1.26 (1.31)	1.33 (1.33)	0.384

**Table 3:** Change in dietary intake of children aged 6 months to 8 years Quetta (*n *263).

Number of servings	Preintervention mean (SD)	Postintervention mean (SD)	*P* value
Number of meals	6.71 (1.72)	6.94 (1.92)	0.025
High starch	3.46 (1.79)	3.58 (1.62)	0.288
Animal protein	0.62 (0.839)	0.70 (0.95)	0.277
Plant protein	1.52 (1.23)	1.26 (1.23)	0.005
Dairy foods	0.78 (1.40)	0.93 (1.67)	0.041
Vegetables	0.36 (0.50)	0.45 (0.62)	0.026
Fruits	0.79 (0.80)	0.89 (0.95)	0.283
Tea	1.97 (1.16)	2.01 (1.17)	0.330
Miscellaneous	0.38 (0.75)	0.33 (0.77)	0.317

## References

[1] https://www.cia.gov/library/publications/the-world-factbook/fields/2091.html.

[2] (2008). *Pakistan Demographic & Health Survey 2006-2007*.

[3] Government of Pakistan, Aga Khan University, Pakistan Medical Research Council Nutrition Wing, UNICEF (2011). *National Nutrition Survey 2011 AKU Report*.

[4] Ali S, Ali SF, Imam AM, Ayub S, Billoo AG (2011). Perception and practices of breastfeeding of infants 0–6 months in an urban and a semi-urban community in Pakistan: a cross-sectional study. *Journal of the Pakistan Medical Association*.

[5] Ansari NB, Rahbar HM, Bhutta ZA, Badruddin SH (2006). Child’s gender and household food insecurity are associated with stunting among young Pakistani children residing in urban squatter settlements. *Food and Nutrition Bulletin*.

[6] Bhutta ZA (2000). Iron and zinc intake from complementary foods: some issues from Pakistan. *Pediatrics*.

[7] Khawar N, Kazmi NR, Barakzai AAL (2002). Etiological factors of malnutrition among infants in two urban slums of Peshawar. *Journal of Postgraduate Medical Institute*.

[8] Kemal AR, Ahmed AM, Nasir ZM, Soomro GY (2001-2002). *National Nutrition Survey 2001-2002*.

[9] Zeman FJ, Hansen RJ (1983). *Clinical Nutrition and Dietetics*.

[10] Rafique G, Khan AK (2010). *Dietary Needs: Children from Birth to Eight Years: Guidelines for Field Workers*.

[11] Rafique G (1986). Guideline for field workers: growth measurement. *How to Weight and Measure Children: Assessing the Nutrition Status of Young Children*.

[12] de Onis M (2006). WHO Child Growth Standards based on length/height, weight and age. *Acta Paediatrica*.

[13] (2007). *WHO Anthro for Mobile Devices Version 2, 2007: Software for Assessing Growth and Development of the World's Children*.

[14] Kilaru A, Griffiths PL, Ganapathy S, Ghosh S (2005). Community-based nutrition education for improving infant growth in rural Karnataka. *Indian Pediatrics*.

[15] Penny ME, Creed-Kanashiro HM, Robert RC, Narro MR, Caulfield LE, Black RE (2005). Effectiveness of an educational intervention delivered through the health services to improve nutrition in young children: a cluster-randomised controlled trial. *The Lancet*.

[16] Bhutta ZA, Soofi S, Cousens S (2011). Improvement of perinatal and newborn care in rural Pakistan through community-based strategies: a cluster-randomised effectiveness trial. *The Lancet*.

[17] Bhutta ZA, Darmstadt GL, Hasan BS, Haws RA (2005). Community-based interventions for improving perinatal and neonatal health outcomes in developing countries: a review of the evidence. *Pediatrics*.

[18] Bhutta ZA, Ahmed T, Black RE (2008). What works? Interventions for maternal and child undernutrition and survival. *Child*.

[19] Jafar TH, Hatcher J, Poulter N (2009). Community-based interventions to promote blood pressure control in a developing country: a cluster randomized trial. *Annals of Internal Medicine*.

[20] Heaver R, Kachondam Y (2002). *Thailand’s National Nutrition Program: Lessons in Management and Capacity Development*.

